# Exposure to environmental chemicals and cancer risk: epidemiological evidence from Japanese studies

**DOI:** 10.1186/s41021-023-00268-3

**Published:** 2023-03-22

**Authors:** Motoki Iwasaki, Hiroaki Itoh, Norie Sawada, Shoichiro Tsugane

**Affiliations:** 1 Division of Epidemiology, National Cancer Center Institute for Cancer Control, 5-1-1 Tsukiji, Chuo-Ku, Tokyo, 104-0045 Japan; 2 Division of Cohort Research, National Cancer Center Institute for Cancer Control, Tokyo, Japan; 3 Department of Epidemiology and Environmental Health, , Juntendo University Faculty of Medicine, Tokyo, Japan; 4 National Institute of Health and Nutrition, National Institutes of Biomedical Innovation, Health and Nutrition, Tokyo, Japan

**Keywords:** Environmental chemicals, Dichlorodiphenyltrichloroethane, Hexachlorocyclohexane, Polychlorinated biphenyls, Per- and polyfluoroalkyl substances, Cadmium, Arsenic, Acrylamide, Cancer risk, Epidemiological study

## Abstract

Exposure to certain chemicals in the environment may contribute to the risk of developing cancer. Although cancer risk from environmental chemical exposure among general populations is considered low compared to that in occupational settings, many people may nevertheless be chronically exposed to relatively low levels of environmental chemicals which vary by such various factors as residential area, lifestyle, and dietary habits. It is therefore necessary to assess population-specific exposure levels and examine their association with cancer risk. Here, we reviewed epidemiological evidence on cancer risk and exposure to dichlorodiphenyltrichloroethane (DDT), hexachlorocyclohexane (HCH), polychlorinated biphenyls (PCBs), per- and polyfluoroalkyl substances (PFASs), cadmium, arsenic, and acrylamide. Japanese are widely exposed to these chemicals, mainly through the diet, and an association with increased cancer risk is suspected. Epidemiological evidence from Japanese studies to date does not support a positive association between blood concentrations of DDT, HCH, PCBs, and PFASs and risk of breast or prostate cancer. We established assessment methods for dietary intake of cadmium, arsenic, and acrylamide using a food frequency questionnaire. Overall, dietary intakes of cadmium, arsenic, and acrylamide were not significantly associated with increased risk of total cancer and major cancer sites in the Japan Public Health Center-based Prospective Study. However, statistically significant positive associations were observed between dietary cadmium intake and risk of estrogen receptor-positive breast cancer among postmenopausal women, and dietary arsenic intake and risk of lung cancer among male smokers. In addition, studies using biomarkers as exposure assessment revealed statistically significant positive associations between urinary cadmium concentration and risk of breast cancer, and between ratio of hemoglobin adducts of acrylamide and glycidamide and risk of breast cancer. Epidemiological studies of general populations in Japan are limited and further evidence is required. In particular, studies of the association of organochlorine and organofluorine compounds with risk of cancer sites other than breast and prostate cancer are warranted, as are large prospective studies of the association between biomarkers of exposure and risk of cancer.

## Background

Environmental factors play an important role in the causation of a majority of human cancers. “Environmental factors” are generally recognized as everything that is not specifically genetic in origin, including tobacco smoking, alcohol consumption, diet and nutrition, infectious agents, radiation, sunlight, exposure to environmental chemicals, and so on. In Japan, the greatest contributing factors to cancer in 2015 were infectious agents and active tobacco smoking, followed by alcohol drinking [1]. Although attributable fraction might be smaller than for infectious agents and tobacco smoking, exposure to certain chemicals in the environment, at home, and at work may contribute to the risk of developing cancer [2]. Environmental chemicals refer to chemical compounds or chemical elements present in air, water, food, soil, dust, or other environmental media such as consumer products. The International Agency for Research on Cancer (IARC) has recognized a number of well-known environment pollutants as posing a carcinogenic hazard in humans, including indoor and outdoor air pollutants, contamination of drinking water by arsenic, and contaminants of soil and food such as dioxin and polychlorinated biphenyls (PCBs) [3–6]. In addition, a large number of environmental chemicals are suspected to be carcinogenic in humans, and further evidence is required [7].

Since the amounts of chemicals in air, water, food, and soil are typically much lower than those in the work environment, cancer risk from environmental chemical exposures among general populations is thought to be low compared to the risk in occupational settings. Nevertheless, even though exposure is likely low, large numbers of people are affected, in accordance with residential area, lifestyle, and dietary habits. It is therefore necessary to assess population-specific exposure levels and to examine their association with cancer risk.

Here, we review epidemiological studies among Japanese, focusing on environmental chemicals to which Japanese are widely exposed, mainly through diet, and are suspected of being associated with increased risk of cancer. This evidence is presented as ‘epidemiological evidence from general populations in Japan’. In addition, we briefly introduce epidemiological evidence commonly seen in Japanese exposed to chemical-contaminated foods or living in polluted areas as background information.

## Organochlorine pesticides

### Dichlorodiphenyltrichloroethane (DDT)

#### Background and overview

One abundant organochlorine contaminant is DDT. Introduced as an insecticide in the 1940s, DDT came into widespread use for insect control in forestry and agriculture and for vector control after World War II. Although most developed countries had banned its use by the early 1980s, some countries still use it for malaria control. Technical-grade DDT predominantly contains *p,pʹ*-DDT and smaller amounts of other compounds such as *o,pʹ*-DDT, *p,pʹ*-DDD (dichlorodiphenyldichloroethane) and *o,pʹ*-DDE (dichlorodiphenyldichloroethylene) [8, 9]. Being both highly lipophilic and resistant to degradation, it is bioaccumulated in the lipid component of biological systems through the food chain [8, 9], and human exposure to DDT and to its metabolite DDE still occurs, mainly through the diet. Indeed, it was widely used in Japan following World War II until the beginning of the 1980s, and residue is still detected in the blood among Japanese [10].

DDT is known as an endocrine disruptor which modulates receptor-mediated effects that can operate in humans [11–13]. Estrogenic effects of *o,pʹ*-DDT and *p,pʹ*-DDT, such as binding and activation of estrogen receptor, have been consistently seen across numerous experimental studies [12, 13]. DDT and its metabolites antagonize the androgen receptor, with *p,pʹ*-DDE being the most potent, and this effect is seen consistently across non-human experimental studies in vivo and in cells from a variety of species, including humans [11]. In addition, there is strong mechanistic evidence that DDT exerts immunosuppression and induces oxidative stress [8]. Accordingly, breast cancer is the most studied cancer site, and more than 40 epidemiological studies have been reported [14]. Interestingly, however, these have shown no association overall between *p,pʹ*-DDE or *p,pʹ*-DDT concentration in blood or adipose tissue and breast cancer risk [14]. For other cancer sites, positive associations have been suggested for liver and testicular cancer and non-Hodgkin lymphoma [15, 16]. IARC evaluated the evidence regarding the carcinogenicity of DDT in humans as limited [8, 17] but, considered together with sufficient evidence for the carcinogenicity of DDT in experimental animals, gave an overall evaluation of probably carcinogenic to humans (Group 2A) in 2015 (Table 1) [8, 17].

**Table 1:** Evaluation of carcinogenic risks to humans by the International Agency for Research on Cancer

Agent	Overall evaluation	Tumour sites (or types) for which there is sufficient evidence in humans	Other sites with limited evidence in humans
Dichlorodiphenyltrichloroethane	Group 2A		Liver, testis, non-Hodgkin lymphoma
Lindane, the γ-isomer of hexachlorocyclohexane	Group 1	Non-Hodgkin lymphoma	
Polychlorinated biphenyls	Group 1	Malignant melanoma	Breast, non-Hodgkin lymphoma
Perfluorooctanoic acid	Group 2B		Testis, kidney
Cadmium and cadmium compounds	Group 1	Lung	Prostate, kidney
Arsenic and inorganic arsenic compounds	Group 1	Lung, skin, urinary bladder	Kidney, liver, prostate
Acrylamide	Group 2A		

### Epidemiological evidence from general populations in Japan

Among epidemiological studies in Japanese, we reported two studies for breast cancer and one for prostate cancer (Table 2) [18–20]. In a nested case–control study within the Japan Public Health Center-based Prospective Study (JPHC Study), measurement of plasma samples from 139 breast cancer cases and 278 controls collected from 1990–1994 found no statistically significant association for *p,pʹ*-DDT and *p,pʹ*-DDE (Table 2) [18]. A stratified analysis by menopausal status showed positive associations in premenopausal but not postmenopausal women, albeit without statistical significance. The second study for breast cancer was a hospital-based case–control study in Nagano Prefecture, Japan, which included 403 pairs recruited from 2001–2005 [19]. No statistically significant association was found for *o,pʹ*-DDT, *p,pʹ*-DDT, or *p,pʹ*-DDE regardless of menopausal status or hormone receptor subtype (Table 2). These findings are in general agreement with a majority of previous studies and meta-analyses including more than 40 studies [14, 21]. This suggests that exposure to DDT during adult life within the range detectable in general populations is not associated with risk of breast cancer. Of interest, however, Cohn et al. reported that higher concentration of *p,pʹ*-DDT during pregnancy was significantly associated with increased risk of breast cancer before age 50 years, with the effect substantially stronger in women exposed before puberty, and strongest for exposure in utero or infancy [22]. On this basis, the possible importance of early-life exposure to DDT has been proposed.

**Table 2:** Summary of epidemiological studies on the association of organochlorines and cancer risk among Japanese

Reference	Study period	Outcome	Design	Study subjects			Exposure assessment	Exposure	Exposure level (median or range among control group)	Category	Odds ratios	95% confidence interval	*P* for trend	Confounding factors
				Definition	Age range	Number of subjects								
Iwasaki et al. [18]	Enrolment 1990–1994, follow-up to 2002	Breast cancer	Nested case– control study	Cases: identified from local hospitals, population-based cancer registries and death certificates, Controls: cohort members matched by age, public health centre area, area, date of blood collection, time of day of blood collection, fasting time at blood collection and menopausal status	40–69 years old at enrolment	139 cases and 278 controls	Plasma samples measured by a gas-chromatography isotope-dilution massspectrometry (GC-IDMS)	*p,pʹ*-DDT (ng/mL)	0.50	Quartile 1	1		0.97	Adjusted for age at menarche, menopausal status and age at menopause, number of births, age at first birth, height, body mass index, and alcohol consumption
									0.89	Quartile 2	0.65	(0.32–1.32)		
									1.36	Quartile 3	0.56	(0.26–1.22)		
									2.24	Quartile 4	0.99	(0.47–2.08)		
								*p,pʹ*-DDE (ng/mL)	2.50	Quartile 1	1		0.25	
									4.83	Quartile 2	1.01	(0.47–2.19)		
									7.58	Quartile 3	1.24	(0.60–2.53)		
									14.41	Quartile 4	1.48	(0.70–3.13)		
								β-HCH (ng/mL)	Not detected	Quartile 1	1		0.34	
									Not detected	Quartile 2	1			
									0.69	Quartile 3	0.84	(0.45–1.57)		
									1.38	Quartile 4	0.74	(0.39–1.39)		
Itoh et al. [19]	Enrolment 2001–2005	Breast cancer	Hospital-based case–control	Cases: histopathologically confirmed, Controls: medical check-up examinees matched by age and area of residence	20–74 years old	403 cases and 403 controls	Serum samples measured by gas chromatography-isotope-dilution high resolution mass spectrometry (GC-ID-HRMS)	*o,pʹ-*DDT (ng/g lipid)	0.90	Quartile 1	1		0.48	Adjusted for total lipid concentration in serum, bodymass index, menopausal status and age at menopause, smoking status, fish consumption, vegetable consumption, family history of breast cancer in a first-degree relative, age at first childbirth, parity, age at menarche, history of breast cancer screening, and history of breast feeding
									1.3	Quartile 2	0.57	(0.25–1.29)		
									2.0	Quartile 3	1.13	(0.53–2.38)		
									4.1	Quartile 4	0.67	(0.30–1.52)		
								*p,pʹ*-DDT (ng/g lipid)	5.6	Quartile 1	1		0.33	
									8.5	Quartile 2	0.58	(0.27–1.25)		
									12.0	Quartile 3	0.99	(0.47–2.07)		
									23.0	Quartile 4	0.58	(0.27–1.25)		
								*p,pʹ*-DDE (ng/g lipid)	160	Quartile 1	1		0.46	
									300	Quartile 2	0.47	(0.24–0.92)		
									490	Quartile 3	0.99	(0.48–2.02)		
									1100	Quartile 4	1.02	(0.46–2.26)		
								β-HCH (ng/g lipid)	26	Quartile 1	1		0.63	
									52	Quartile 2	0.81	(0.39–1.72)		
									82	Quartile 3	0.72	(0.31–1.69)		
									160	Quartile 4	1.04	(0.43–2.52)		
								Total PCBs (ng/g lipid)	110	Quartile 1	1		0.008	
									160	Quartile 2	0.79	(0.36–1.72)		
									200	Quartile 3	0.57	(0.28–1.15)		
									290	Quartile 4	0.33	(0.14–0.78)		
Sawada et al. [20]	Enrolment 1990–1994, follow-up to 2005	Prostate cancer	Nested case– control study	Cases: identified from local hospitals, population-based cancer registries and death certificates, Controls: cohort members matched by age, public health centre area, area, date of blood collection, time of day of blood collection, and fasting time at blood collection	40–69 years old at enrolment	201 cases and 402 controls	Plasma samples measured by gas chromatography-isotope-dilution high resolution mass spectrometry (GC-ID-HRMS)	*o,pʹ-*DDT (ng/g lipid)	< 2.5	Quartile 1	1		0.61	Adjusted for smoking status, alcohol intake, marital status, body mass index, and intake of green tea and miso soup
									2.5–4.2	Quartile 2	1.39	(0.79–2.44)		
									4.3–7.6	Quartile 3	1.29	(0.71–2.34)		
									7.7	Quartile 4	1.04	(0.54–2.03)		
								*p,pʹ*-DDT (ng/g lipid)	< 24	Quartile 1	1		0.45	
									24–40	Quartile 2	1.51	(0.87–2.63)		
									41–63	Quartile 3	0.92	(0.50–1.70)		
									64	Quartile 4	1.00	(0.52–1.92)		
								*p,pʹ*-DDE (ng/g lipid)	< 560	Quartile 1	1		0.65	
									560–939	Quartile 2	1.00	(0.60–1.66)		
									940–1599	Quartile 3	0.89	(0.52–1.53)		
									1600	Quartile 4	0.90	(0.52–1.54)		
								β-HCH (ng/g lipid)	< 200	Quartile 1	1		0.05	
									200–319	Quartile 2	0.89	(0.52–1.50)		
									320–519	Quartile 3	0.85	(0.50–1.46)		
									520	Quartile 4	0.56	(0.30–1.01)		
								Total PCBs (ng/g lipid)	< 319	Quartile 1	1		0.90	
									319–447	Quartile 2	1.06	(0.63–1.79)		
									448–668	Quartile 3	0.84	(0.49–1.46)		
									669	Quartile 4	0.97	(0.51–1.87)		

*DDT* Dichlorodiphenyltrichloroethane, *DDE *Dichlorodiphenyldichloroethylene, *HCH *Hexachlorocyclohexane, *PCBs *Polychlorinated biphenyls

We also conducted a nested case–control study of prostate cancer within the JPHC Study, using plasma samples collected from 201 cases and 402 matched controls between 1990 and 1994 [20]. No statistically significant association was seen for *o,pʹ*-DDT, *p,pʹ*-DDT, or *p,pʹ*-DDE regardless of stage at diagnosis of prostate cancer (localized or advanced) (Table 2). This result is consistent with a meta-analysis based on studies that measured serum DDT concentration in the general population—although a positive association was found in studies based on occupational exposure, it was not statistically significant [23]. Similarly to the case with breast cancer, this indicates no elevated risk of prostate cancer within the range detectable among general populations.

### Hexachlorocyclohexane (HCH)

#### Background and overview

Lindane, the γ-isomer of HCH, has been extensively used for insect control in agriculture and for treatment of human ectoparasites. Technical-grade HCH, containing approximately 60–70% α-HCH, 5–12% β-HCH, 10–40% γ-HCH, 6–10% δ-HCH, and 3–4% ε-HCH, has reportedly been used as an insecticide although only γ-HCH has insecticidal properties [8]. Technical-grade HCH was banned for production and use in the United States in 1976, but may still be used in other countries in small quantities [24]. Although worldwide use of HCH declines, it has been measured in food, air, surface water, groundwater, sediment, soil, fish, wildlife, and humans as a consequence of biological persistence. Relatively many epidemiological studies have provided consistent evidence of a positive association between mostly occupational exposure to lindane and the risk of non-Hodgkin lymphoma [15, 16], and it was consequently evaluated as carcinogenic to humans (Group 1) in 2015 (Table 1) [8, 17]. Meanwhile, several epidemiological studies of the association of lindane or HCH isomers (β-HCH) measured in blood with the risk of breast, prostate, or testicular cancer reported inconsistent results [8, 23]. Although the blood concentration of β-HCH might be a surrogate marker of exposure to lindane, exposure to β-HCH can occur through the diet and through contact with other environment media. The blood concentration of β-HCH might therefore not reflect exposure to lindane, and this is likely one reason for these inconsistent findings.

### Epidemiological evidence from general populations in Japan

We measured β-HCH concentration in plasma or serum samples in a nested case–control study for breast or prostate cancer within the JPHC Study and in a hospital-based case–control study in Nagano Prefecture, Japan, as described above (Table 2) [18–20]. Overall, β-HCH concentration was not significantly associated with increased risk of breast cancer in either study [18, 19], while an inverse association was found for prostate cancer, albeit without statistical significance [20].

### PCBs

#### Background and overview

PCBs are a class of aromatic compounds comprising 209 congeners, each containing one to ten chlorine atoms attached to a biphenyl nucleus. They were predominantly used as dielectric fluids (in transformers and electric capacitors) and as additives for pesticides, flame retardants, insulators, paints, glues and printing inks [5, 25]. Similarly to organochlorine pesticides, they are now classified as persistent organic pollutants (POPs) and are ubiquitously present in the environment worldwide due to persistence and bioaccumulation, though their production was banned in most countries by the 1980s. Exposure in the general population today is mainly from eating contaminated foods or breathing contaminated air [5, 25].

PCBs or their metabolites have been shown to exert genotoxic effects, immune suppression, inflammatory responses, and endocrine effects via a number of mechanisms [5, 26–28]. Low-chlorinated PCBs involved in oxidative metabolism may produce oxidative stress and genotoxicity [28]. Meanwhile, highly chlorinated PCBs activate aryl hydrocarbon receptor (AhR) and the constitutive androstane and pregnane xenobiotic receptor (CAR/PXR). In particular, 12 PCB congeners that have a strong affinity for the AhR are referred to as “dioxin-like PCBs”. Activation of the AhR is one of the key events linked to carcinogenesis mediated by dioxin-like compounds [27]. In addition, they interact with nuclear steroid hormone receptor and can act as estrogen agonists or antagonists [26].

Based on studies documenting an increased risk of cutaneous malignant melanoma—mostly occupational cohort studies—PCBs were classified by IARC in 2013 as carcinogenic to humans (Group 1) (Table 1) [5, 29]. Positive associations were suggested for non-Hodgkin lymphoma and breast cancer, but evaluation found limited evidence for the carcinogenicity of PCBs in humans. Notable epidemiological evidence in Japan came from a cohort study of Yusho patients, who experienced a mass food poisoning incident from cooking oil accidentally contaminated with PCBs, polychlorinated dibenzofurans (PCDFs), and other dioxin-related compounds in 1968: a recent 50-year follow-up study of these patients showed a significant increase in standardized mortality ratio for all cancers, and for lung cancer in men and liver cancer in women [30]. Blood concentrations of some PCB congeners were approximately 3–4 times higher than in the normal control group even 35 years after exposure [31]. In addition to studies in subjects with relatively high exposure levels, future studies should also clarify the effect of exposure to PCBs on cancer risk at blood concentrations within the range detectable among general populations in Japan.

### Epidemiological evidence from general populations in Japan

We have reported two studies in general populations in Japan (Table 2) [19, 20]. In the hospital-based case–control study in Nagano Prefecture, mentioned above, measurement of 41 PCB peaks showed a significant association of serum lipid-adjusted concentration of total PCBs with decreased risk of breast cancer (Table 2) [19]. An inverse association was observed regardless of hormone receptor subtype or menopausal status, and half of 34 individual congeners were associated with decreased risk of breast cancer. Moreover, selected PCB congeners were categorized into group 1A (weak phenobarbital inducers, estrogenic, not persistent), group 1B (weak phenobarbital inducers, persistent), group 2A (potentially antiestrogenic and immunotoxic, dioxin-like, moderately persistent), group 2B (potentially antiestrogenic and immunotoxic, limited dioxin activity, persistent), and group 3 (phenobarbital, cytochrome P450 1A [CYP1A] and cytochrome P450 2B [CYP2B] inducers, biologically persistent) according to their structural, biological and pharmacokinetics properties as proposed by Wolff et al. [26, 32]. Statistically significant inverse associations were observed for groups 1B, 2B, and 3, whereas no statistically significant association was seen for group 1A or 2A. Eventually, we did not observe a positive association between the serum concentration of PCBs and risk of breast cancer among Japanese women. According to a meta-analysis based on case–control or cohort studies using measured PCB concentration in biological samples, a statistically significant positive association was not found for total PCBs, but was found for groups 2 and 3 [33]. This is inconsistent with our Japanese study, which showed an overall inverse association. The reason for this discrepancy is unclear but might be partly explained by the difference in concentrations of individual congeners and their composition across studies.

The second study was the nested case–control study of prostate cancer within the JPHC Study mentioned above [20]. We measured 41 PCB peaks and found no statistically significant association of prostate cancer risk with total PCBs, individual PCBs, or for PCBs grouped according to the definition of Wolff et al. (Table 2) [32]. In addition, no statistically significant differences were seen for total PCBs according to stage at diagnosis (localized or advanced). Although few previous studies are available, their findings are inconsistent [34, 35]. For example, a nested case–control study within a population-based cohort from Norway found no statistically significant association for total PCBs and most individual PCBs, but did find a significant inverse association for PCB 44 [34]. In contrast, a case-cohort study within the Korean Cancer Prevention Study-II showed statistically significant positive associations for moderately or highly chlorinated PCBs, total PCBs, and group 3 by the definition of Wolff et al. [32, 35].

## Per- and polyfluoroalkyl substances (PFASs)

### Background and overview

PFASs are synthetic organofluorine chemical compounds that have been used since the 1950’s in a variety of industrial and commercial applications, such as firefighting foams, non-stick cookware, waterproof clothing, cosmetics, and paper coating used in food packaging [36, 37]. Due to extreme resistance to biodegradation, they are highly persistent in the environment and are classified as POPs. They can bioaccumulate in humans with serum elimination half-lives ranging from about 3 to 8 years and have been detected in blood among most populations as consequence of their widespread use [38, 39]. The major sources of exposure for most of the general population are contamination of drinking water; food, mainly seafood, including transfer of PFASs from food packaging; some consumer products; and household dust [36, 40, 41]. The production, use, import and export of perfluorooctane sulfonate (PFOS), perfluorooctanoic acid (PFOA), and perfluorohexane sulfonate (PFHxS) have been controlled by the Stockholm Convention on Persistent Organic Pollutants since 2009, 2019, and 2022, respectively.

Of the thousands of PFASs currently in use, the two most studied are PFOS and PFOA, owing to their relatively higher environmental levels compared to other PFASs. At this point, only PFOA has been evaluated for carcinogenicity by IARC, which classified it as a possible human carcinogen (Group 2B) based on limited evidence for testicular and kidney cancer from human and animal studies, in addition to moderate evidence for carcinogenic mechanisms (Table 1) [42, 43]. This evaluation referred to epidemiological studies showing positive associations with the risk of testicular and kidney cancer among highly exposed subjects working or living near PFAS production plants [44–46]. Regarding the mechanism of PFOA carcinogenesis, some studies indicated that its induction of oxidative stress may induce indirect DNA damage, but that direct genotoxicity is unlikely [42]. Animal studies indicated a potential role of peroxisome proliferator-activated receptor alpha (PPARα) activation, which is a crucial factor in the regulation of lipid metabolism and inflammation [47]. Moreover, endocrine disrupting properties have been shown, and several studies have suggested estrogenic and anti-androgenic activities [48, 49].

#### Epidemiological evidence from general populations, including Japanese evidence

In addition to epidemiological studies among highly exposed populations, a population-based prospective cohort study in the US found a statistically significant positive association of prediagnostic serum PFOA concentration, which was comparable with the general population, with the risk of renal cell carcinoma [50]. Moreover, an increasing number of studies have examined associations with the risk of breast cancer, given that a potential mechanism of carcinogenicity is their endocrine disrupting properties [21]. Findings have been inconsistent, however: Mancini et al. reported a statistically significant positive association between serum PFOS concentration and risk of hormone receptor-positive breast cancer among non-occupationally exposed postmenopausal French women [51] while Hurley et al. observed no overall significant association between serum PFASs concentration and risk of breast cancer in a nested case–control study within the California Teachers Study, despite a statistically significant inverse association of serum perfluoroundecanoic acid (PFUnDA) and PFHxS concentration with risk of hormone receptor-negative breast cancer [52].

Recently, we measured serum concentrations of 20 PFAS congeners using samples from a hospital-based case–control study in Nagano Prefecture, Japan, and found a statistically significant inverse association of 17 of 20 PFAS congeners and risk of breast cancer [53]. Table 3 shows the results for total PFOS (n-PFOS, 1 m-PFOS, 3 m-PFOS, 4 m-PFOS, 5 m-PFOS, and 6 m-PFOS) and total PFOA (n-PFOA and 6 m-PFOA). Although we distinguished branched PFAS isomers from linear isomers (the prefix “n-” indicates a linear isomer, whereas a prefix starting with a number indicates a branched isomer), similar associations were observed (data not shown). Since only a few epidemiological studies have been reported to date, further accumulation of evidence is desirable [21].

**Table 3:** Summary results for the association of per- and polyfluoroalkyl substances (PFASs) with cancer risk among Japanese

Reference	Study period	Outcome	Design	Study subjects			Exposure assessment	Exposure	Exposure level (median or range among control group)	Category	Odds ratios	95% confidence interval	*P* for trend	Confounding factors
				Definition	Age range	Number of subjects								
Itoh et al. [53]	Enrolment 2001–2005	Breast cancer	Hospital-based case–control	Cases: histopathologically confirmed, Controls: medical check-up examinees matched by age and area of residence	20–74 years old	401 cases and 401 controls	Serum samples measured by in-port arylation gas chromatography-negative chemical ionization-mass spectrometry (GC-NCI-MS)	Total PFOS (ng/mL)	7.63	Quartile 1	1		0.0001	Adjusted for body mass index, height, menopausal status and age at menopause, age at first childbirth, family history of breast cancer, smoking status, strenuous physical activity in the past five years, moderate physical activity in the past five years, age at menarche, number of births, breastfeeding duration, alcohol intake, isoflavone intake, education level, serum total concentrations of polychlorinated biphenyls, fish and shellfish intake, vegetable intake, and calendar year of blood sampling
									12.2	Quartile 2	0.38	(0.18–0.82)		
									16.27	Quartile 3	0.31	(0.14–0.69)		
									24.67	Quartile 4	0.15	(0.06–0.39)		
								Total PFOA (ng/mL)	3.18	Quartile 1	1		0.001	
									4.71	Quartile 2	0.37	(0.19–0.73)		
									6.46	Quartile 3	0.39	(0.18–0.84)		
									9.31	Quartile 4	0.20	(0.08–0.51)		
*PFOS *Perfluorooctane sulfonate, *PFOA *Perfluorooctanic acid														

## Cadmium

### Background and overview

Cadmium is found at low concentrations in the Earth's crust, mainly as the sulfide in zinc-containing mineral deposits, and is widely but sparsely distributed through natural activities, such as volcanic activity, weathering and erosion, and river transport. It has also been widely dispersed into the environment through mining, smelting and refining of nonferrous metals, industrial emissions, and incineration of municipal waste (especially cadmium-containing batteries and plastics). Other than occupational exposure, the major source of cadmium exposure in general populations is foods, in addition to tobacco smoking, since cadmium contained in soil and water can be taken up by certain crops and aquatic organisms and accumulate in the food chain [54].

Although a well-known health effect of chronic exposure to cadmium is renal dysfunction, IARC classified cadmium and cadmium compounds as carcinogenic to humans (Group 1) based on sufficient evidence for an increase in lung cancer risk, mostly from studies in occupational settings (Table 1) [3, 55]. Moreover, positive associations have been suggested for kidney and prostate cancer. Several mechanisms that potentially contribute to cadmium-induced carcinogenesis have been identified [3, 56–60]: cadmium alters DNA repair and tumor-suppressor proteins, leading to chromosomal damage and genomic instability [58, 59]; induces alterations in epigenetic and signal transduction processes, which may contribute to the deregulation of cell growth; and shows estrogenic properties in both in vitro and in vivo studies [56, 57].

Studies in cadmium-polluted areas in Japan showed that urinary excretion concentration of β2-microglobulin as a marker of cadmium toxicity was significantly associated with increased risk of cancer mortality, but the small number of cancer deaths did not allow further detailed analysis [61]. A study of the cadmium-polluted Jinzu River basin in Toyama, Japan, however, revealed significantly increased mortality risk for cancer from total, renal, and uterus among exposed women with proteinuria, glucosuria, and glucoproteinuria based on a review of historical records [62]. Meanwhile, the effect of cadmium exposure on cancer occurrence among Japanese in non-polluted areas has been of particular interest considering that mean cadmium level via food in a general population in Japan (26 µg/ day) [63] is higher than that in general populations in China (10 µg/day) [64] and Sweden (15 µg/day) [65].

### Epidemiological evidence from general populations in Japan

**Fig. 1:**
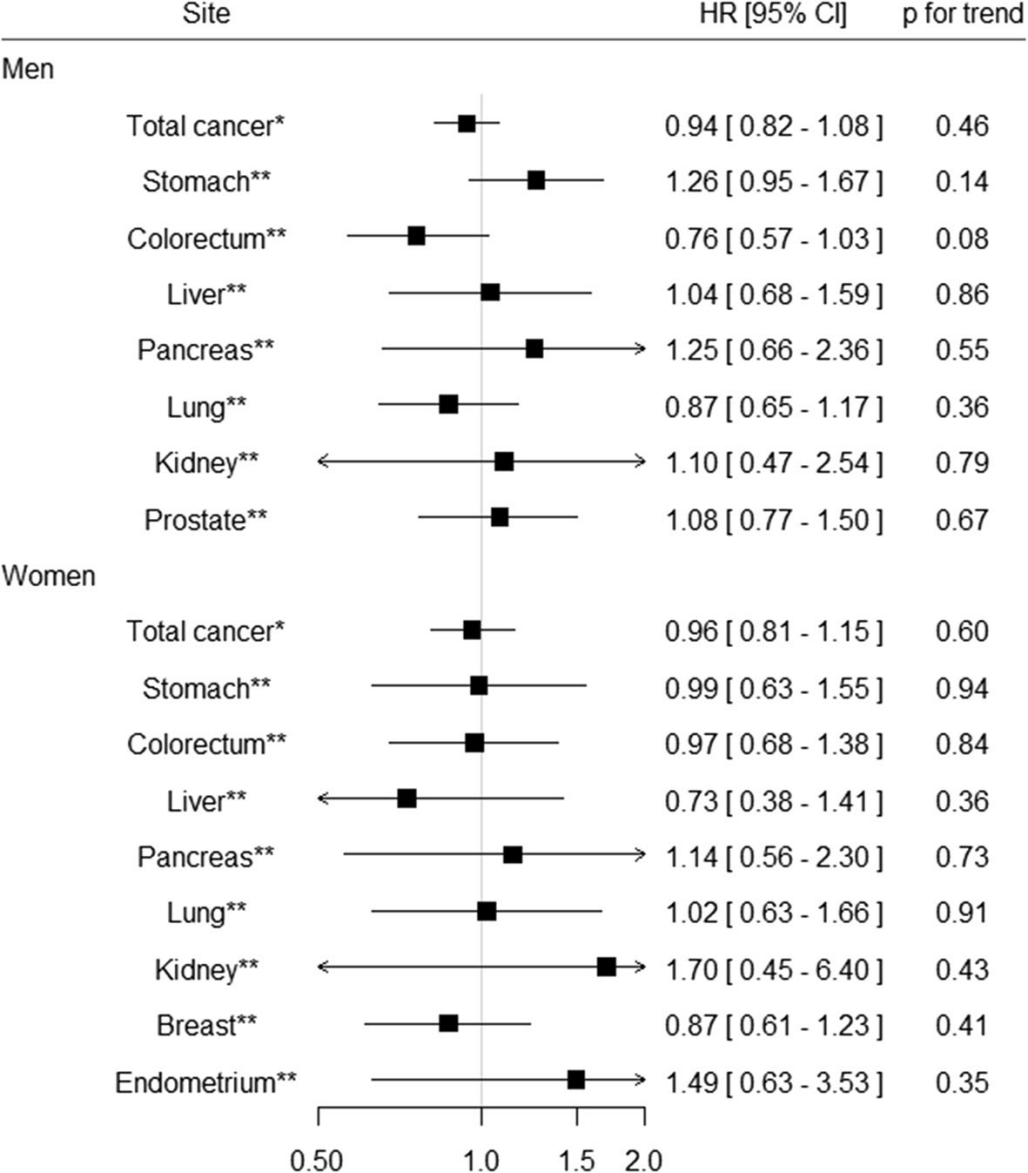
Summary results for the association between dietary cadmium intake and cancer risk in the JPHC Study * Quartile category ** Tertile category Data from Sawada et al. [66]. Adjusted HRs (95% CIs) for the highest versus lowest category are shown CI, confidence interval; HR, hazard ratio; JPHC Study, Japan Public Health Center-based Prospective Study

We therefore developed a practical method for assessing dietary cadmium intake using a food frequency questionnaire (FFQ) used for the JPHC Study, and evaluated the validity of the FFQ in estimating intake by comparing 24-h urinary excretion of cadmium with dietary cadmium intake estimated from the FFQ [66]. Spearman correlation coefficients between these variables were 0.38 in men and 0.45 in women, indicating that the FFQ is reasonably valid for assessing cadmium intake in epidemiological studies. We then examined the association of dietary cadmium intake with risk of total cancer and site-specific cancers using data from the 5-year follow-up survey of the JPHC Study [66]. No statistically significant association was observed for total cancer or site-specific cancer in 9 years of follow-up data for 90,383 Japanese men and women aged 45–74 years (Fig. 1). In contrast, a cohort study in general populations living in non-cadmium-polluted areas (Ishikawa and Chiba Prefecture) found that urinary cadmium concentration was significantly associated with increased risk of total cancer and pancreatic cancer mortality for women but not men, based on 19-year follow-up of 1107 men and 1697 women [67].

The estrogenic properties of cadmium have focused attention on their role in the etiology of breast cancer [68]. As described, dietary cadmium intake was not associated with the risk of breast cancer in the JPHC Study (Fig. 1 and Table 4) [66]. We also investigated the association between dietary cadmium intake and risk of breast cancer in a hospital-based case–control study in Nagano Prefecture, Japan. Although no statistically significant association was observed for breast cancer overall (Table 4), higher cadmium intake was significantly associated with increased risk of estrogen receptor-positive breast cancer among postmenopausal women [69]. Adjusted OR (95% CI) for the highest versus lowest tertile was 1.94 (1.04–3.63) and the trend test was also statistically significant (*p* = 0.032). Furthermore, a hospital-based case–control study in Gifu Prefecture, Japan measured urinary cadmium concentration using spot urine samples collected from 153 breast cancer cases and 431 matched control subjects [70]. A statistically significant positive association was found between urinary cadmium concentration and risk of breast cancer (Table 4). Adjusted ORs (95% CI) for the highest versus lowest tertile were 6.05 (2.90–12.62) for all subjects and 4.60 (2.67–10.2) among never smokers. Considering the findings from studies using urinary biomarkers [68, 70], additional evidence from prospective studies with a large sample size are warranted.

**Table 4:** Summary results for the association between cadmium and breast cancer risk among Japanese

Reference	Study period	Design		Study subjects		Exposure assessment	Exposure	Exposure level (median or range among control group)	Category	Relative risk	95% confidence interval	*P* for trend	Confounding factors
			Definition	Age range	Number of subjects								
Sawada et al. [66]	Enrolment 1995–1998, follow-up to 2006	Population-based cohort	Residents in 10 public health center areas in Japan	45–74 years old at enrolment	402 breast cancer cases among 48,351 women	Food frequency questionnaire	Dietary intake (ug/day)	19.2	Tertile 1	1		0.41	Adjusted for age, area, body mass index, smoking status, frequency of alcohol intake, leisure-time physical activity, intake of meat, soybean, vegetable, and fruit, menopausal status, and use of exogenous female hormones
								24.9	Tertile 2	1.16	(0.87–1.54)		
								32.3	Tertile 3	0.87	(0.61–1.23)		
Itoh et al. [69]	Enrolment 2001–2005	Hospital-based case–control	Cases: histopathologically confirmed, Controls: medical check-up examinees matched by age and area of residence	20–74 years old	390 cases and 390 controls	Food frequnecuy questionnaire	Dietary intake (ug/day)	21.4	Tertile 1	1		0.39	Adjusted for menopausal status, moderate physical activity in the past five years, smoking status, family history of breast cancer, number of births, isoflavone intake, vegetable intake, and total energy intake
								26.2	Tertile 2	1.19	(0.79–1.79)		
								31.5	Tertile 3	1.23	(0.76–2.00)		
Nagata et al. [70]	Enrolment 2000–2002	Hospital-based case–control	Cases: histopathologically confirmed, Controls: examinees of breast cancer mass screening matched by age, menopausal status, and period of urine sampling	25–70 + years old	153 cases and 431 controls	Spot urine samples measured by flameless atomic absorption spectrometry	Urinary cadmium (ug/g creatinine)	< 1.674	Tertile 1	1		< 0.01	Adjusted for age, years of education, age at menarche, number of births, age at first birth, body mass index, smoking status, alcohol intake, and family history of breast cancer among first-degree relatives
								1.674–2.620	Tertile 2	2.25	(1.17–4.37)		
								> 2.620	Tertile 3	6.05	(2.90–12.62)		

## Arsenic

### Background and overview

Arsenic is used in industrial processes such as nonferrous smelting, wood preservation, glass manufacturing, production and application of arsenic-based pesticides, and electronics. Inhalation is the primary route of exposure to arsenic in the workplace [3]. Arsenic is widely distributed throughout the Earth’s crust, and is a known contaminant of many groundwater sources worldwide via dissolution or desorption of minerals. In addition to naturally occurring groundwater contamination, it can also occur as a consequence of mining activities, use of arsenic-based pesticides and herbicides, industrial processes, and irrigation. In countries where inorganic arsenic is present at high levels in the groundwater, drinking water is major route of exposure, whereas food is usually the major contributor in areas where arsenic levels are not naturally high [71].

Regarding the health effects of long-term exposure, IARC classified arsenic and inorganic arsenic compounds as carcinogenic to humans (Group 1) based on epidemiological studies which showed increased risk of lung, skin, and urinary bladder cancer due to exposure to arsenic through inhalation in the workplace or drinking water contaminated with high levels of inorganic arsenic (Table 1) [3, 55]. Moreover, a positive association has been observed between exposure to arsenic and inorganic arsenic compounds and risk of kidney, liver, and prostate cancer. Several mechanisms by which arsenic and inorganic arsenic compounds induce carcinogenesis have been proposed, including induction of oxidative DNA damage and DNA-repair inhibition, aneuploidy, gene amplification, and epigenetic alterations leading to altered gene expression and genomic instability [3, 60].

In Japan, a historical cohort study was conducted in an arsenic-polluted area in which well water was polluted by liquid waste containing inorganic arsenic from a dye factory [72]. Results showed a significant increase in mortality for lung and urinary tract cancer among residents who drank well water containing a high concentration of arsenic (≥ 1 ppm). Meanwhile, the Japanese Water Supply Law and Ordinance restricts arsenic concentration in drinking water to less than 0.01 mg/L, and most Japanese are therefore exposed to arsenic via foods, particularly seafood and seaweeds [73]. Although the arsenic in seafood and seaweeds is usually in the form of organic compounds, which are known to have low toxicity, arsenosugars detected in seaweeds are metabolized to dimethylarsinic acid in humans, which is more toxic than arsenosugars [74, 75]. In addition, the edible seaweed *hijiki* (Hizikia fusiforme) contains inorganic arsenic, and concerns over a potential effect on cancer risk have been raised given its relatively common consumption among seaweeds by Japanese [76].

### Epidemiological evidence from general populations in Japan

**Fig. 2:**
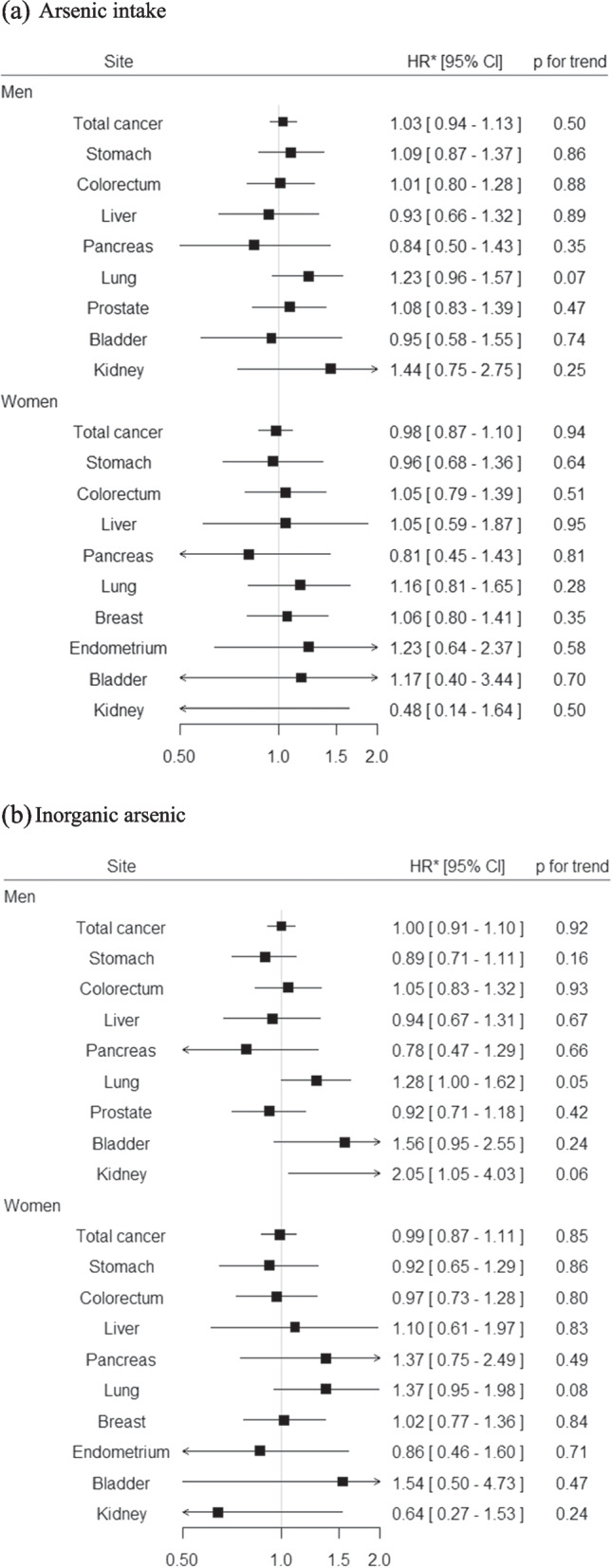
Summary results for the association between dietary arsenic intake and cancer risk in the JPHC Study. **a** Arsenic intake (**b**) Inorganic arsenic Data from Sawada et al. [73] *Adjusted HRs (95% CIs) for the highest versus lowest quartile category are shown CI, confidence interval; HR, hazard ratio; JPHC Study, Japan Public Health Center-based Prospective Study

We developed a validated method for estimating dietary arsenic intake based on the FFQ used in the JPHC Study [73]. From a validation study in a subsample of subjects in the JPHC Study which compared intake between the FFQ and dietary records (DRs), Spearman’s rank correlation coefficients for arsenic and inorganic arsenic were 0.30 and 0.33 in men and 0.15 and 0.19 in women, respectively. The contributions of seafood, *hijiki*, seaweeds, rice, and vegetables to total arsenic intake were 32%, 28%, 20%, 16%, and 1%, respectively, while the contributions of *hijiki*, rice, seaweeds, seafood, vegetables, and fruits to inorganic arsenic intake were 50%, 35%, 5%, 4%, 3%, and 2%, respectively. We investigated the association of dietary arsenic intake with risk of total and site-specific cancer based on 10.9 years of follow-up data for 90,378 Japanese men and women aged 45–74 years in the JPHC Study [73]. Overall, we found no statistically significant association between total arsenic and inorganic arsenic intake and risk of total cancer for both men and women, respectively (Fig. 2 (a) and (b)).

For site-specific cancers, no statistically significant association was observed for total arsenic intake among both men and women (Fig. 2 (a)) [73]. Meanwhile, we found positive associations of inorganic arsenic intake with risk of lung cancer for both men and women, and risk of kidney cancer for men (Fig. 2 (b)). Of interest, subgroup analysis by smoking status revealed a statistically significant interaction of total arsenic intake and smoking status in relation to the risk of lung cancer among men: a statistically significant positive association was found for ever smokers while a statistically significant inverse association was seen for never smokers (data not shown). A similar result was observed for inorganic arsenic intake among men, although without statistical significance. On the other hand, a positive association was found for never smokers but a test for interaction was not statistically significant among women. This observed interaction among men is consistent with studies characterized by a high level of arsenic exposure [72, 77, 78]. On the other hand, the reason for the discrepant results between men and women is less clear, although possible explanations include the small number of smokers and relatively low validity of estimated arsenic intake from the FFQ among women.

## Acrylamide

### Background and overview

Acrylamide is a chemical compound produced industrially mainly as a precursor to polyacrylamides, which have a variety of uses such as water-soluble thickeners and flocculation agents. In 1994, IARC classified acrylamide as probably carcinogenic to humans (Group 2A) based on sufficient evidence in experimental animals for the carcinogenicity of acrylamide (Table 1) [79]. Mechanistic evidence has shown that acrylamide and its metabolite glycidamide form DNA and hemoglobin adducts and that acrylamide induces gene mutation and chromosomal aberrations [80, 81]. In addition to acrylamide-induced genotoxicity, hormonal pathways have been hypothesized, particularly given that tumorgenicity by acrylamide was found in rat endocrine and mammary gland [80, 81].

Prior to 2002, exposure to acrylamide was thought to primarily occur in occupational settings or through tobacco smoke. The discovery in 2002 that some cooked foods contain acrylamide, however, has raised concerns about potential health effects in the general population [82]. The major pathway by which acrylamide is formed in foods is through the Maillard reaction during food cooking at temperatures higher than > 120 °C [82]. The content of acrylamide in foods varies widely, depending on the food matrix and food processing method. As a consequence of different dietary habits and cooking methods across countries, dietary exposure to acrylamide and foods contributing to acrylamide intake might differ across populations, which in turn indicates the necessity of population-specific studies to assess dietary exposure level and its association with cancer risk.

### Epidemiological evidence from general populations in Japan

We established a validated method for estimating dietary acrylamide intake from the FFQ used for the JPHC Study [83]. Deattenuated correlation coefficients for energy-adjusted dietary acrylamide intake between DRs and the FFQ ranged from 0.37 to 0.54, which indicates its suitability for use in epidemiological studies. Mean acrylamide intake from DRs was about 7 µg/day, and 0.12 µg/kg body weight/day, which was less than in European populations (12 to 48 µg/day in 27 areas of 10 European countries within the European Prospective Investigation into Cancer and Nutrition (EPIC) study) [84, 85]. The main contributing food groups from DRs in the JPHC Study were beverages, confectioneries, vegetables, potatoes and starches, and cereals [83]. In contrast, the primary contributing foods in European countries were potato-based foods (eg, fried potato), wheat-based products (eg, biscuits), and coffee [84, 85].

**Fig. 3:**
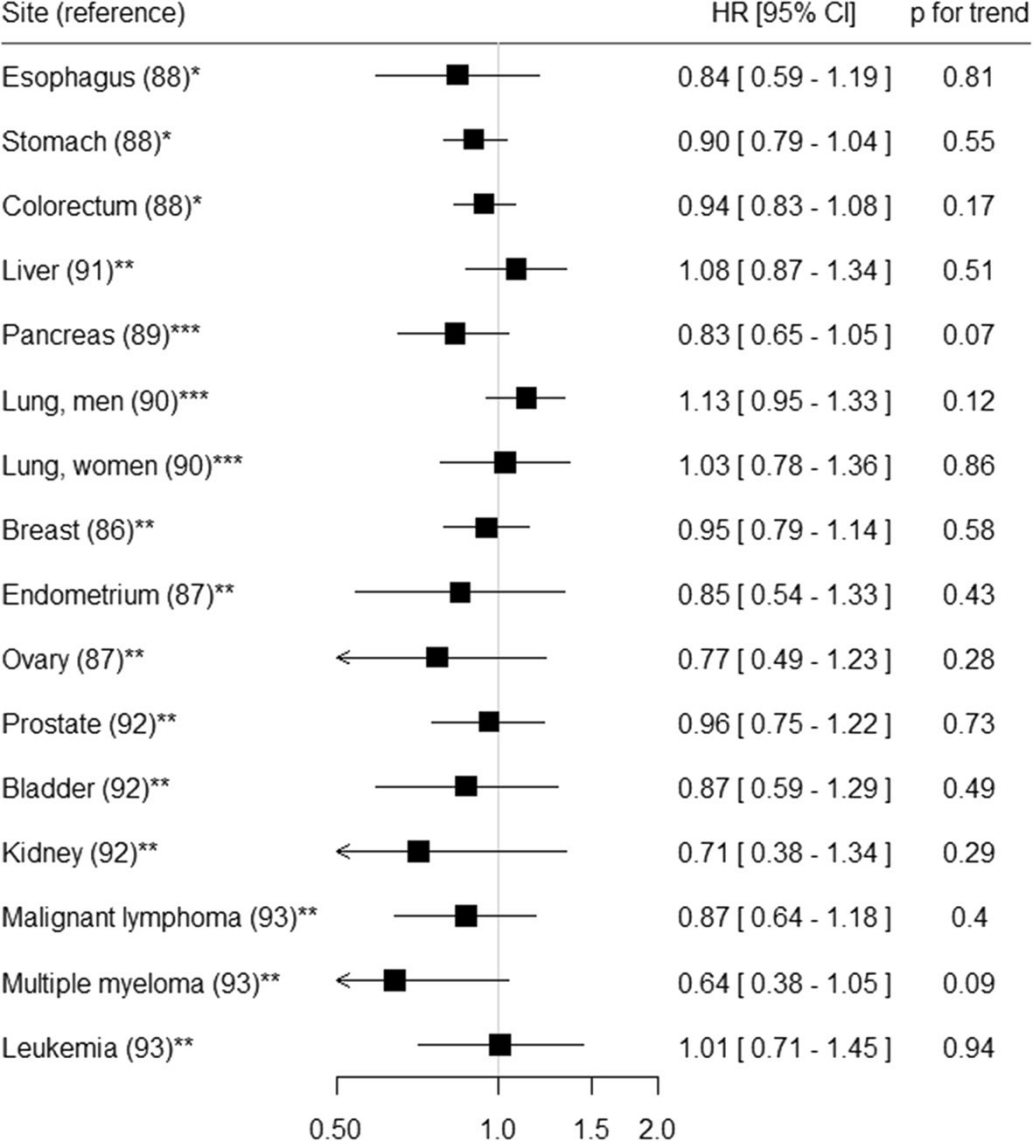
Summary results for the association between dietary acrylamide intake and cancer risk in the JPHC Study * Quintile category ** Tertile category *** Quartile category Adjusted HRs (95% CIs) for the highest versus lowest category are shown CI, confidence interval; HR, hazard ratio; JPHC Study, Japan Public Health Center-based Prospective Study

We examined the association of dietary acrylamide intake with risk of site-specific cancers using data from the 5-year follow-up survey of the JPHC Study [86–93]. No statistically significant association was observed for any cancer site (Fig. 3). These findings are in general agreement with recent meta-analyses [94, 95]. Meanwhile, we recently measured hemoglobin adducts of acrylamide and glycidamide (HbAA and HbGA) in erythrocytes collected from 125 breast cancer cases and 250 controls in a nested case–control study within the JPHC Study [96]. We found no statistically significant positive association for either HbAA or HbGA, but a positive association between HbGA/HbAA ratio and risk of breast cancer. Adjusted OR (95% CI) for the highest versus lowest tertile was 2.19 (1.11–4.31) and the trend test was also statistically significant (*p* = 0.027). Given that acrylamide is primarily metabolized by phase I enzymes such as cytochrome P450 2E1 (CYP2E1) to the epoxide metabolite, glycidamide, which is likely to play an important role in the carcinogenicity [80, 81], HbGA/HbAA ratio reflects individual differences in susceptibility to acrylamide exposure and might be a biomarker for acrylamide-related genotoxic exposure [97].

## Conclusions

We reviewed epidemiological studies of DDT, HCH, PCBs, PFASs, cadmium, arsenic, and acrylamide, to which Japanese populations are widely exposed. DDT, HCH, PCBs, and PFASs were detected in blood samples from general populations in Japan. Epidemiological evidence did not support positive associations between blood concentration of DDT, HCH, PCBs, and PFASs and risk of breast and prostate cancer. We established assessment methods for the dietary intake of cadmium, arsenic, and acrylamide using an FFQ. Overall, dietary intake of cadmium, arsenic, and acrylamide was not significantly associated with increased risk of total cancer and major cancer sites in the JPHC Study. However, a statistically significant positive association was observed between dietary cadmium intake and risk of estrogen receptor-positive breast cancer among postmenopausal women, and between dietary arsenic intake and risk of lung cancer among male smokers. In addition, studies using biomarkers as exposure assessment revealed statistically significant positive associations between urinary cadmium concentration and risk of breast cancer, and between HbGA/HbAA ratio and risk of breast cancer. Since the number of epidemiological studies in general populations in Japan is limited, further accumulation of evidence is required, with particular focus on the following: the association of organochlorine and organofluorine compounds with risk of cancer sites other than breast and prostate cancer; and large prospective studies of the association between biomarkers of exposure and risk of cancer. For the latter, collaboration among relevant researchers to ensure that epidemiological studies incorporate precise biologic sample-based exposure assessment will be essential.

## Data Availability

Not applicable.

## Abbreviations

AhR: Aryl hydrocarbon receptor
CAR: Constitutive androstane receptor
CI: Confidence interval
CYP1A: Cytochrome P450 1A
CYP2B: Cytochrome P450 2B
CYP2E1: Cytochrome P450 2E1
DDD: Dichlorodiphenyldichloroethane
DDE: Dichlorodiphenyldichloroethylene
DRs: Dietary records
DDT: dichlorodiphenyltrichloroethane
EPIC: European Prospective Investigation into Cancer and Nutrition
FFQ: Food frequency questionnaire
HbAA: Hemoglobin adducts of acrylamide
HbGA: Hemoglobin adducts of glycidamide
HCH: Hexachlorocyclohexane
IARC: International Agency for Research on Cancer
JPHC Study: Japan Public Health Center-based Prospective Study
OR: Odds ratio
PCBs: Polychlorinated biphenyls
PCDFs: Polychlorinated dibenzofurans
PFASs: Per- and polyfluoroalkyl substances
PFHxS: Perfluorohexane sulfonate
PFOA: Perfluorooctanoic acid
PFOS: Perfluorooctane sulfonate
PFUnDA: Perfluoroundecanoic acid
PHC: Public health center
POPs: Persistent organic pollutants
PPARα: Peroxisome proliferator-activated receptor alpha
PXR: Pregnane xenobiotic receptor
